# Retraction: Exosome-Derived miR-486-5p Regulates Cell Cycle, Proliferation and Metastasis in Lung Adenocarcinoma via Targeting NEK2

**DOI:** 10.3389/fbioe.2021.681918

**Published:** 2021-03-26

**Authors:** Frontiers Editorial Office

The journal retracts the April 8, 2020 article cited above.

Following publication, the authors contacted the Editorial Office to request the retraction of the cited article, stating that they discovered a fault in their experimental design that rendered the conclusions unreliable. An investigation was conducted in accordance with our established procedures that confirmed this; therefore, the article has been retracted.

The authors concur with the retraction and sincerely regret any inconvenience this may have caused to the reviewers, editors, and readers of Frontiers in Bioengineering and Biotechnology.

